# Bidirectional Within-Family Effects of Restrictive Mediation Practices and Adolescents’ Problematic Social Media Use

**DOI:** 10.1007/s10964-024-01990-z

**Published:** 2024-05-03

**Authors:** Suzanne M. Geurts, Helen G. M. Vossen, Regina J. J. M. Van den Eijnden, Ina M. Koning

**Affiliations:** 1 Interdisciplinary Social Science, Utrecht University, Padualaan 14, 3584 CH Utrecht, The Netherlands; 2 Education and Pedagogy, Utrecht University, Heidelberglaan 1, 3584 CS Utrecht, The Netherlands; 3 Educational and Family Studies, VU Amsterdam, Van der Boechorststraat 7, 1081 BT Amsterdam, The Netherlands

**Keywords:** Adolescents, Problematic social media use, Parental restrictive mediation, Directionality

## Abstract

Much remains unknown about whether restrictive mediation is an effective parenting strategy to prevent or reduce problematic social media use among adolescents. Therefore, this study examined bidirectional within-family effects between two restrictive mediation practices (rule-setting and reactive restrictions) and problematic social media use using random-intercept cross-lagged panel modeling. Three-wave survey data collected among Dutch adolescents (T1: *N* = 1928, *M*_age_ = 13.31 years, *SD* = 0.91, 43.3% girl) with a 1 year-interval were used. Results showed that within-family changes in problematic social media use symptoms predicted subsequent within-family changes in perceived parental restrictive mediation. More specifically, an increase in symptoms predicted a decrease in rule-setting and an increase in reactive restrictions 1 year later. Within-family changes in perceived parental restrictive mediation practices did not predict within-family changes in problematic social media use symptoms, suggesting that the relation is unidirectional. However, concluding that limiting adolescents’ Internet use is ineffective to prevent problematic social media use would be premature. Future research should investigate whether it may be an effective parenting strategy for a certain subgroup of adolescents or under certain circumstances.

## Introduction

Little is known about the effectiveness of restrictive mediation in limiting adolescents’ problematic social media use. The limited research available on the link between restrictive mediation and problematic social media use is inconclusive (see for review: Lukavská et al., 2022; Vossen et al., 2024). This ambiguity may stem from a lack of differentiation between rule-setting and reactive restrictions in most studies. The few studies that did make this distinction showed differing associations of problematic social media use with these two types of restrictive mediation (Geurts et al., 2022; Koning et al., 2018). Besides, almost all studies used a cross-sectional design making it impossible to draw conclusions about the direction of the association. Therefore, this study aims to examine the longitudinal bidirectional relation of both rule-setting and reactive restrictions with adolescents’ problematic social media use. This will be done on the within-family level to gain insights into the dynamic within-family processes instead of relative differences between families (Hamaker et al., 2015).

### Adolescents’ Problematic Social Media Use

According to a recent, representative Dutch report, approximately one in three secondary school students almost constantly uses social media to keep in touch with friends (Boer et al., 2022a). Despite the numerous opportunities it offers, the pervasiveness of social media in adolescents’ lives has led to a growing concern among parents about the potential negative consequences of the use of these platforms. As these platforms are designed to keep the user’s attention as long as possible, it is not surprising that parents are concerned about adolescents losing control over their use such that it leads to impairment in daily functioning. The prevalence of this so-called problematic social media use among adolescents differs per country, ranging from 3 to 16% in Europe (Boer et al., 2022b). Recent research showed that problematic social media use may promote attention problems, impulsivity (e.g., Boer et al., 2020a), anxiety (e.g., Chang et al., 2022), cybervictimization (e.g., Boer et al., 2021) and depressive symptoms (e.g., Raudsepp & Kais, 2019), and may lower life satisfaction (e.g., Van den Eijnden et al., 2018). Because of the possible detrimental impact of problematic social media use on adolescents’ mental health, there is a societal call for concrete advice and guidance for parents on how to manage their children’s online engagement to prevent the development of addictive-like symptoms and, instead, help their children establishing a healthy relationship with social media (Montag et al., 2024).

### Parental Restrictive Mediation

The parental mediation theory postulates that parents use a variety of strategies to mitigate the possible negative effects of media use (Clark, 2011). Qualitative research revealed that parents often apply restrictive mediation out of concern for their children becoming addicted to social media (Gür & Türel, 2022; Wang & Chen, 2022). Restrictive mediation refers to regulating children’s media use by setting boundaries and limitations. In this study, two types of restrictive mediation practices are distinguished, namely, rule-setting and reactive restrictions. Internet-specific rule-setting is defined as the degree to which parents allow access to the Internet in terms of the amount of time, location of use, time of day of use and content (Koning et al., 2018). Reactive restrictions toward Internet use refer to reactive, in the moment, attempts of parents to limit their children’s Internet use.

### Testing Bidirectional Within-Family Processes

Previous research has shown that adolescents who report more problematic social media use symptoms tend to report less parental rule-setting and more reactive restrictions (Geurts et al., 2022). However, these correlations lack insight into temporal ordering. As much as it is likely that parents influence their child’s behavior through parenting practices, it is likely that parents react to the behavior of their child by adjusting their parenting practices (Sameroff, 2009). Understanding the temporal ordering is essential to appropriately target intervention efforts (Vrolijk et al., 2023). For example, if restrictive mediation precedes problematic social media use, interventions can focus on empowering parents to implement effective restrictive mediation practices. On the other hand, if problematic social media use precedes restrictive mediation practices rather than the other way around, interventions may need to address other factors that do contribute to problematic social media use, such as psychological vulnerabilities or peer influences (Xu et al., 2021). The one study that did examine potential reciprocal effects over time only showed a (marginally) significant protective effect of Internet-specific rule-setting on problematic social media use among girls (Koning et al., 2018). Yet, this study’s findings reflect relative differences between families that do not necessarily translate to dynamic processes that occur within families (Boele et al., 2020). Answering questions like: “Does a change in parental Internet-specific rule-setting for a particular adolescent predict a subsequent change in problematic social media use symptoms of that adolescent?” is required for making causal inferences that can inform interventions about effective parenting practices.

### Internet-Specific Rule-Setting and Problematic Social Media Use

Parents who implement rules to restrict their children’s Internet use do this with the intention to limit negative consequences such as problematic use. For example, rules on the amount of time of use per day and on taking the smartphone to the bedroom when going to sleep are expected to limit the opportunity to (further) develop problematic use of social media (Geurts et al., 2022; Koning et al., 2018). Following a similar line of reasoning, loosening of rules may provide adolescents with greater freedom and opportunities to engage in excessive social media use. Therefore, it is expected that a decrease in Internet-specific rules is related to an increase in problematic social media use symptoms over time.

As much as adolescent behavior may be influenced by their parents, parents’ behavior can be influenced by the child (e.g., Leung, 2021). The protection motivation theory (PMT) describes the conscious, reasoned processes underlying the adoption of protective behaviors (Rogers, 1975). Although PMT is mainly used to predict individuals’ behaviors that protect themselves, the theory has also been used to predict behaviors that are aimed at protecting others, including parental restrictive mediation (e.g., Hwang et al., 2017). According to PMT, protective behavior is predicted by two cognitive processes: threat appraisal and coping appraisal. Threat appraisal involves an individual’s perception of the severity of and susceptibility to the situation. Coping appraisal is an individual’s assessment of their ability to handle the perceived threat effectively through protective behaviors (i.e., efficacy). Using this theory, one could argue that an increase in problematic social media use symptoms is related to either an increase or a decrease in Internet-specific rule-setting. On the one hand, as an increase in symptoms means an increase in the severity of the problem, parents may perceive an enhanced threat which may drive parents to setting (stricter) rules. On the other hand, when parents have applied rules in the past, an increase in symptoms may lower parents’ expectation that setting rules will be effective in removing the perceived threat, and, therefore, decrease their rule-setting.

### Reactive Restrictions Toward Internet Use and Problematic Social Media Use

Reactive restrictions can be expected to frustrate the adolescent’s desire to keep using social media (Koning et al., 2018). More specifically, these restrictions can be perceived by the adolescent as a threat to their freedom. The psychological reactance theory posits that a perceived threat to an individual’s freedom induces psychological reactance (Brehm, 1993). Psychological reactance is an unpleasant arousal that motivates individuals to re-establish the threatened freedom. In this way, the “forbidden fruit” becomes even more desirable. Thus, parental reactive restrictions may elicit such psychological reactance, resulting in engaging in excessive use when having the opportunity to compensate for the perceived loss of freedom by the imposed restrictions (e.g., Meeus et al., 2019). In line with this theory, previous research has shown that restrictive mediation can have the opposite effect as intended (such as viewing restricted content), because it elicits psychological reactance (White et al., 2015). Another line of thinking is that when parents frequently intervene in their children’s Internet use by saying that they must stop, they take away opportunities for adolescents to practice with controlling their online behavior by themselves (Koning et al., 2018). If adolescents become unable to control their social media use themselves, this may contribute to more problematic use.

In turn, parental reactive restrictions may be triggered reactively when parents perceive that their child is engaging in social media use excessively. The transactional model emphasizes the reciprocal and interactive processes between parents and children (Sameroff, 2009). This model, which is underpinned with empirical evidence (see for review Sameroff & Mackenzie, 2003), posits that parenting practices and children’s behavior influence each other over time, creating a continuous feedback loop. An adolescent displaying problematic social media use symptoms may elicit parental reactive restrictions toward Internet use, which, in turn, may influence the development of symptoms of problematic social media use.

## Current Study

The existing body of research concerning the association between restrictive mediation and adolescents’ problematic social media use is limited, inconclusive and primarily comprises cross-sectional studies. Therefore, the current study investigates the bidirectional within-family effects between two forms of restrictive mediation (rule-setting and reactive restrictions) and problematic social media use symptoms over time. Based on the idea that loosening rules about the amount, location and moment of Internet use increases the opportunities to engage in excessive social media use, the first hypothesis is that a decrease in parental Internet-specific rule-setting is related to an increase in problematic social media use symptoms. Based on the protection motivation theory, the second hypothesis is that an increase in symptoms is related to either an increase or a decrease in parental Internet-specific rule-setting. The third hypothesis, derived from the psychological reactance theory, states that an increase in parental reactive restrictions toward Internet use is related to an increase in problematic social media use symptoms. Based on the transactional model, the fourth hypothesis is that an increase in symptoms is related to an increase in parental reactive restrictions toward Internet use.

## Methods

### Procedure

Three waves of data from a larger longitudinal research project entitled Digital Youth project (Van den Eijnden et al., 2018) were used. In this project, online surveys were administered by researchers or research assistants during school hours at both urban and suburban secondary schools across the Netherlands. Selection of schools was guided by the principal investigator’s network including key figures working in schools. Participation was voluntary and anonymous. Information letters were distributed prior to data collection to explain the aim and nature of the project, and to provide students or their parents the opportunity to refuse participation. Besides, during data collection participants were told that they could withdraw from participation at any time. The Ethics Review Board of the Faculty of Social and Behavioral Sciences approved the study procedures (FETC16-076 Eijnden). For the current study, data collected in February and March of 2016, 2017 and 2018 were used; here referred to as T1, T2 and T3.

### Participants

In total, 1928 adolescents from eight different schools participated at T1, of which 1420 (73.65%) participated at T2 and 712 (36.93%) at T3. Drop out was mainly due to complete classes or schools dropping out because they were not able to organize participation in the later waves (e.g., unable to schedule time during class hours for participation in the survey). At T2, one school dropped out and at T3 another two schools. Individual students dropped out either due to leaving the school or absence on the day of data collection. At T1, participants were aged between 10 and 16 years (*M* = 13.31, SD = 0.91) and 43.3% were girls. Regarding education, adolescents enrolled in higher level education (higher general and pre-university: 31.1%) appeared underrepresented compared the populations of Dutch secondary school students (45.8%) (VO Raad, 2015–2016). The study sample appeared representative in terms of ethnicity; 74.6% had a Dutch background (self and parents born in the Netherlands) (Central Bureau for Statistics, 2024). Compared to those who dropped out, adolescents who remained in the study at T2 were more likely to be girls (*χ*^2^ = 6.28, *p* = 0.012), to be in higher level education (*χ*^2^ = 31.286, *p* < 0.001), to have a Dutch background (*χ*^2^ = 13.964, *p* < 0.001) and to report less problematic social media use symptoms at T1 (*t*(795.970) = −4.13, *p* < 0.001). Adolescents who remained in the study at T3 were more likely to be in higher level education (*χ*^2^ = 409.019, *p* < 0.001), to have a Dutch background (*χ*^2^ = 41.732, *p* < 0.001) and to report less problematic social media use symptoms (*t*(1922) = −3.57, *p* < 0.001) and more parental Internet-specific rule-setting (*t*(1808) = 4.98, *p* < 0.001) at T1.

### Measures

#### Problematic social media use

Problematic social media use was measured using the Social Media Disorder scale (Van Den Eijnden et al., 2016). The scale consists of nine items measuring nine symptoms of addiction to social media (preoccupation, withdrawal, tolerance, persistence, displacement, conflict, deception, escape and problems). Each item included a dichotomous response scale (*yes* or *no*) for reporting whether the adolescent experienced the particular symptom in the past year. An example item is “During the past year, have you regularly found that you can’t think of anything else but the moment that you will be able to use social media again?” (preoccupation). Higher scores on the scale indicated more symptoms. Because of the dichotomous items, internal consistency was assessed by calculating the tetrachoric ordinal alpha (Gadermann et al., 2012). Ordinal alphas were 0.83 (T1), 0.85 (T2) and 0.86 (T3).

#### Internet-specific rule-setting

Internet-specific rules were measured using five items with a five-point response scale ranging from *never* (1) to *very often* (5). Adolescents were, for instance, asked to indicate to what extent they were allowed to bring their smartphones to their bedroom when going to sleep at night on regular school days (Koning et al., 2018). To ensure that a higher score reflected more rule-setting, all items were reverse coded. Cronbach’s alphas were 0.77 (T1), 0.82 (T2) and 0.83 (T3).

#### Reactive restrictions toward Internet use

Reactive restrictions toward Internet use were measured using four items asking adolescents how often their parents react in a certain way when they want to use/keep on using the Internet (Koning et al., 2018). Example items are “that you have to turn off your computer, tablet or smartphone” and “that you have a certain time (left) to use the Internet or play games (e.g., 5 min)”. Adolescents replied on a five-point scale ranging from (*almost*) *never* (1) to *more than five times a day* (5). Higher scores on the scale indicated more reactive restrictions. Cronbach’s alphas were 0.84 (T1), 0.85 (T2) and 0.89 (T3).

### Data Analyses

Analyses were performed in Mplus version 8.8. To examine the bidirectional within-family relations between the two restrictive mediation practices and adolescents’ problematic social media use, a multiple indicator random-intercept cross-lagged panel model (RI-CLPM) was applied following the modeling steps as proposed by Mulder & Hamaker (2021). A multiple indicator RI-CLPM is an extension of the basic RI-CLPM (Hamaker et al., 2015) that separates the variance of each variable into a between-family and within-family part on the latent rather than the observed level (Mulder & Hamaker, 2021). By doing so, measurement error is taken into account which results in less biased estimates of the associations between constructs. For the between-family part, a random intercept is included for each latent construct that represents the time-invariant differences between families. Because of a non-significant negative variance of the random intercept for problematic social media use – indicating little to no stable between-person differences in this variable (each person fluctuates around the same sample mean over time) – the random intercept factor for problematic social media use was removed (Mulder & Hamaker, 2021). The random intercepts for rule-setting and reactive restrictions were correlated. The within-family part uses within-family centered variables that captures individuals’ deviations from their own expected score. The expected score is based on an individual’s random intercept and the sample mean. Structural paths are specified on the within-family level, including cross-lagged paths, autoregressive paths and within-wave correlations. Cross-lagged paths estimate the extent to which an individual’s deviation from the expected score of one variable are predicted by deviations from the expected score of another variable on the previous measurement wave. Autoregressive paths estimate to what extent deviations from one’s own expected score at one measurement wave carry over to the next wave. Within-wave correlations estimate the extent to which changes occur simultaneously within the same measurement wave. To obtain the most parsimonious model, the model fit of an unconstrained model in which all paths were freely estimated was compared to three constrained models: (1) a model with all autoregressive paths constrained to be equal over time, (2) a model with all cross-lagged paths constrained to be equal over time and (3) a model with all autoregressive and cross-lagged paths constrained to be equal over time. A reduction in CFI of not more than 0.010 and an increase in RMSEA by not more than 0.015 means that the model does not fit significantly worse (Chen, 2007) implying that the constraints are tenable, and the effects are time-invariant (Mulder & Hamaker, 2021). To determine the magnitude of cross-lagged effects, benchmarks as outlined in the work by Orth et al. (2022) were used with standardized regression coefficients of 0.03, 0.07 and 0.12 indicating small, moderate and large effects, respectively.

Running a multiple indicator RI-CLPM requires establishing longitudinal measurement invariance. Therefore, before testing the structural model, three models that impose successive restrictions on factor loadings and thresholds/intercepts were identified and compared. First, a RI-CLPM was identified with factor loadings freely estimated over time for the within-family factors to test configural invariance (invariant factor structure across waves). Second, a RI-CLPM was identified with factor loadings constrained equal over time to assess weak factorial invariance (invariant relationships of the items with the latent factor across waves). Third, a RI-CLPM was identified with, in addition to the factor loadings, the item intercepts/thresholds constrained equal over time to assess strong factorial invariance (invariant average scores on the items across waves). Again, changes in CFI (ΔCFI ≥ −0.010) and RMSEA values (ΔRMSEA ≥ 0.015) were used to evaluate whether model fit of the constrained model did not become significantly worse than the model fit of the less constrained model, implying that the equality constraints hold. At least weak factorial invariance should be established to meaningfully interpret the structural paths of the RI-CLPM on the within-family level (Mulder & Hamaker, 2021).

WLSMV estimator in combination with theta parametrization was used because of the dichotomous indicators of problematic social media use. Full information maximum likelihood was used to handle missing data. This approach uses all available data to estimate the parameters which results in less bias compared to using listwise deletion of cases with missing values (Enders & Bandalos, 2001).

## Results

### Descriptive Statistics

Means, standard deviations and correlations for problematic social media use, Internet-specific rule-setting and reactive restrictions toward Internet use are displayed in Table 1.

**Table 1:** Means, standard deviations and correlations between all study variables

Variable	*M*	SD	1	2	3	4	5	6	7	8	9
1. PSMU T1	1.29	1.58	–								
2. PSMU T2	1.17	1.51	0.44***	–							
3. PSMU T3	1.09	1.47	0.37***	0.52***	–						
4. Rule-setting T1	3.22	1.03	−0.16***	−0.07*	−0.02	–					
5. Rule-setting T2	2.99	1.09	−0.14***	−0.09**	0.05	0.55***	–				
6. Rule-setting T3	2.65	1.08	−0.11**	−0.04	−0.01	0.54***	0.58***	–			
7. Reactive restrictions T1	1.71	0.77	0.18***	0.11***	0.20***	0.23***	0.22***	0.19***	–		
8. Reactive restrictions T2	1.62	0.75	0.07**	0.21***	0.17***	0.23***	0.20***	0.28***	0.42***	–	
9. Reactive restrictions T3	1.53	0.73	0.11**	0.16***	0.21***	0.20***	0.21***	0.28***	0.38***	0.46***	–

Spearman’s Rho was used for correlations with PSMU. Pearson correlation was used for correlations with rule-setting and reactive restrictions

*p < 0.05, **p ≤ 0.01, ***p ≤ 0.001

### Longitudinal Measurement Invariance

Table 2 shows the model fit indices for each step of testing measurement invariance over time. The weak and strong factorial invariance models did not fit the data significantly worse than the configural model, providing support for strong factorial invariance. Hence, the measurement model based on strong factorial measurement invariance was specified to estimate the RI-CLPM.

**Table 2:** Model fit indices for longitudinal measurement invariance testing

Model	CFI	∆CFI	TLI	RMSEA	∆RMSEA
Configural	0.957	–	0.953	0.013	–
Weak factorial	0.959	0.002	0.956	0.013	–
Strong factorial	0.955	−0.004	0.952	0.014	0.001

*CFI* comparative fit index, *∆CFI* change in comparative fit index, *TLI* Tucker–Lewis index, *RMSEA* root mean square error of approximation, *∆RMSEA* change in root mean square error of approximation

### RI-CLPM with Rule-Setting, Reactive Restrictions and Problematic Social Media Use

To obtain the most parsimonious model, the RI-CLPMs with constrained autoregressive and/or cross-lagged paths over time were compared to the RI-CLPM in which the autoregressive and cross-lagged paths were freely estimated. The unconstrained RI-CLPM showed good model fit (CFI = 0.930, TLI = 0.929, RMSEA = 0.016). Model fit slightly improved when either the autoregressive paths (CFI = 0.931, TLI = 0.930, RMSEA = 0.016) or the cross-lagged paths (CFI = 0.931, TLI = 0.931, RMSEA = 0.016), or both the autoregressive and cross-lagged paths (CFI = 0.933, TLI = 0.932, RMSEA = 0.016) were constrained to be time-invariant. On grounds of parsimony and the latter model having the best model fit, the model with equality constraints on the autoregressive as well as cross-lagged paths was selected as the final model. Based on the above, it can be concluded that the dynamic processes were invariant over the time span of the study.

Table 3 shows the results of the RI-CLPM. The within-family cross-lagged effects demonstrated large effects of problematic social media use at T1 and T2 on restrictive mediation practices at T2 and T3, respectively, but no effects the other way around. More specifically, adolescents reporting more problematic social media use symptoms than usual, reported an increase in parental reactive restrictions and a decrease in parental rule-setting 1 year later. However, adolescents who experienced more rule-setting or reactive restrictions than usual did not report a change in problematic social media use symptoms a year later. In addition, large effects of reactive restrictions at T1 and T2 on rule-setting at T2 and T3, respectively, were found. Adolescents who experienced more reactive restrictions toward their Internet use than usual experienced an increase in Internet-specific rule-setting 1 year later. Rule-setting did not predict reactive restrictions over time.

**Table 3:** Standardized parameter estimates of the final RI-CLPM for rule-setting, reactive restrictions and PSMU

	*β*	SE	*p*
Cross-lagged effects			
PSMU T1 → rule-setting T2	−0.250	0.077	**0.001**
PSMU T2 → rule-setting T3	−0.264	0.082	**0.001**
Rule-setting T1 → PSMU T2	0.067	0.062	0.284
Rule-setting T2 → PSMU T3	0.079	0.075	0.297
PSMU T1 → reactive restrictions T2	0.253	0.065	**0.000**
PSMU T2 → reactive restrictions T3	0.270	0.067	**0.000**
Reactive restrictions T1 → PSMU T2	−0.037	0.051	0.467
Reactive restrictions T2 → PSMU T3	−0.036	0.050	0.467
Rule-setting T1 → reactive restrictions T2	0.086	0.070	0.224
Rule-setting T2 → reactive restrictions T3	0.106	0.088	0.231
Reactive restrictions T1 → rule-setting T2	0.212	0.074	**0.004**
Reactive restrictions T2 → rule-setting T3	0.215	0.078	**0.006**
Autoregressive effects			
PSMU T1 → PSMU T2	0.819	0.046	**0.000**
PSMU T2 → PSMU T3	0.833	0.071	**0.000**
Rule-setting T1 → rule-setting T2	0.161	0.099	0.102
Rule-setting T2 → rule-setting T3	0.197	0.127	0.120
Reactive restrictions T1 → reactive restrictions T2	0.068	0.075	0.362
Reactive restrictions T2 → reactive restrictions T3	0.070	0.079	0.376
Within-wave correlations			
PSMU T1 ↔ rule-setting T1	−0.314	0.063	**0.000**
PSMU T2 ↔ rule-setting T2	−0.077	0.096	0.420
PSMU T3 ↔ rule-setting T3	0.178	0.145	0.221
PSMU T1 ↔ reactive restrictions T1	0.354	0.043	**0.000**
PSMU T2 ↔ reactive restrictions T2	0.429	0.068	**0.000**
PSMU T3 ↔ reactive restrictions T3	0.285	0.124	**0.022**
Rule-setting T1 ↔ reactive restrictions T1	0.000	0.104	0.997
Rule-setting T2 ↔ reactive restrictions T2	0.023	0.077	0.767
Rule-setting T3 ↔ reactive restrictions T3	0.204	0.073	**0.005**
Random intercept variances			
Rule-setting	0.747	0.088	**0.000**
Reactive restrictions	0.131	0.015	**0.000**
Between-person correlations			
Rule-setting ↔ reactive restrictions	0.516	0.064	**0.000**

Significant *p* values are displayed in bold

*β* standardized beta coefficient, *SE* standard error, *p* *=* *p-* value

Regarding within-family autoregressive effects, only significant effects were found for problematic social media use from both T1–T2 and T2–T3. That is, adolescents reporting an increase in problematic social media use symptoms (relative to their expected score) are likely to still report an increase in problematic social media use symptoms at the subsequent measurement wave (relative to their expected score). In other words, it indicates that increased problematic social media use symptoms are persistent over the time span of the study.

Within-wave correlations showed that, within-families, an increase in problematic social media use symptoms was associated with an increase in reactive restrictions both at T1 and T2. Besides, at T1, an increase in problematic social media use symptoms was associated with a decrease in rule-setting. At T3, an increase in reactive restrictions was associated with an increase in rule-setting.

The random intercept factors for rule-setting and reactive restrictions correlated significantly indicating that, at the between-family level, adolescents who reported more parental rule-setting also reported more parental reactive restrictions. Due to the absence of stable between-person differences in problematic social media use, no random intercept factor was included for this variable, precluding the assessment of between-family associations with problematic social media use.

## Discussion

Previous research on the relation between parental restrictive mediation and adolescents’ problematic social media use has produced inconsistent results, largely relying on cross-sectional data. Consequently, little is understood about the bidirectional effects at play. Seeking to enhance our understanding of the possible effectiveness of restrictive mediation, the current study delved into the longitudinal bidirectional effects between two forms of restrictive mediation (rule-setting and reactive restrictions) and adolescents’ problematic social media use at the within-family level. The analyses revealed unidirectional effects of adolescents’ social media behavior on restrictive mediation, where increased problematic social media use symptoms predicted decreased rule-setting, but increased reactive restrictions.

### Adolescent-Driven Processes

Since, on the basis of the protection motivation theory, an increase in problematic social media use symptoms could relate to either an increase or a decrease in parental Internet-specific rule-setting, this relation was investigated in an exploratory way. The analyses revealed that adolescents whose symptoms increased, subsequently reported less parental rule-setting 1 year later. Possibly, parents become more permissive over time in allowing access to the Internet in response to the development of problematic social media use. This response could stem from parents’ sense of resignation or frustration when previous rules appear ineffective in controlling their child’s social media use. When rule-setting appears ineffective, this may lower parents’ beliefs in their ability to influence their child’s social media use (i.e., parental self-efficacy). Previous research showed that parents are less likely to apply restrictive mediation when they have lower self-efficacy (Chang et al., 2019; Glatz et al., 2018). A similar reaction of parents is found for other adolescent risk behaviors such as alcohol use; parents became less strict in rule-setting once their child started drinking alcohol (more frequently) (Glatz et al., 2012; Koning et al., 2013).

In addition, the results showed that, as expected, adolescents whose symptoms increased, subsequently reported that their parents applied more reactive restrictions toward their Internet use. Thus, although the previously discussed finding could imply that parents give up on setting clear rules, this finding suggests that parents may react more impulsively by applying corrective measures more frequently in response to signs of problematic social media use. This seems congruent with the literature showing that adolescent involvement in problematic gaming and alcohol use predicts poorer parenting such as more reactive parenting, letting go of rules and lower quality of communication (Koning et al., 2013, 2018). Parental concerns about their child’s wellbeing may cause this reactive approach as these reactive restrictions may be an attempt to exert direct control over their child’s online behavior (Wilson et al., 2011). Besides, for adolescents who develop (more) problematic social media use symptoms, it becomes more and more difficult to stop using social media on their own. A logical consequence is that parents need to intervene more frequently.

Although it seems highly plausible that our finding reflects an actual increase in parental reactive restrictions, it is also important to consider the role of adolescents’ subjective experience. Adolescents showing problematic social media use are preoccupied with social media and experience discomfort and anxiety when having no access to the platforms (Griffiths et al., 2014). Therefore, perhaps, when adolescents’ problematic social media use symptoms increase, they may perceive their parents’ restrictions as more intrusive and strict, leading them to report experiencing more reactive restrictions even though their parents did not increase their intervening.

### Parent-Driven Processes

Contrary to the study’s hypotheses, changes in Internet-specific rule-setting and reactive restrictions did not predict changes in problematic social media use. Thus, these findings suggest that, among 10–16-year-olds, none of the two restrictive mediation practices are effective in preventing or reducing problematic social media use symptoms, nor do they promote the development of problematic social media use. While parental rules might be effective in reducing screen time (Fam et al., 2023), they may not address the underlying factors (e.g., unsatisfied psychological needs) driving problematic social media use (Geurts et al., 2023a). However, concluding that limiting adolescents’ Internet use is ineffective in preventing or reducing this problematic use would be premature, as it may be an effective parenting strategy for a certain subgroup of adolescents or under certain circumstances. For example, the effect of rules could depend on whether they are set in an autonomy-supportive or controlling way as this may influence the extent to which adolescents adhere to or internalize parental rules (Chng et al., 2015; Valkenburg et al., 2013). Moreover, whereas older adolescents may feel that parental rules about their Internet use are unwarranted as they strive for greater autonomy in decision-making over certain behaviors, younger adolescents may perceive parental rules as more legitimate (Babskie & Metzger, 2018). To better understand the influence of restrictive mediation on adolescents’ problematic social media use, it is important for future research to consider possible moderators.

Moreover, it is also important to keep in mind that this study is done among adolescents aged 10–16 years while most children in the Netherlands get their first smartphone, and thus access to social media, from the age of 9 (Burgering, 2022). Therefore, the findings do not rule out the possibility that setting rules in an earlier stage – before social media become a central part of adolescents’ life – could have a preventive effect. This would be in line with research on alcohol use showing that strict rules about alcohol consumption had a protective effect, particularly among adolescents who had not started drinking alcohol yet (Koning et al., 2014; Van Der Vorst et al., 2006). Longitudinal data starting from an earlier age are needed to investigate whether making strict agreements from the very first moment children are using social media platforms can be protective.

### Limitations

The current study has some shortcomings that should be acknowledged. First, as already touched upon when interpreting the findings; it is important to consider that the study relied on adolescent reports. This may have introduced reporting bias. For example, adolescents may not always provide accurate self-reports of their problematic social media use, because of socially desirable answering tendencies, or being unaware of or unable to recall certain symptoms (Austermann et al., 2021; Geurts et al., 2023b). Yet, parent-reports are also biased and, when examining the effects of parenting behaviors on child development, it has been argued that children’s subjective experiences are more meaningful than parent self-reports or observations (e.g., Bögels & Van Melick, 2004). Nevertheless, it may be interesting to see if current findings hold when using parent or multi-informant reports.

Second, a substantial proportion of the participants (42.3–47.8%) reported experiencing no symptoms of problematic social media use. As variables cannot be specified as count variables in a RI-CLPM, the estimated values in this study may therefore be slightly biased.

Third, although in a RI-CLPM the within-family effects are controlled for all possible time-invariant covariates such as personality traits and gender, the model does not account for time-varying factors. Therefore, it cannot be ruled out that factors such as adolescents’ age and parent–child conflict may have contributed to the observed effects.

Fourth, it remains to be examined whether current findings can be generalized to other cultures. Effects of restrictive mediation on problematic social media use might be different in collectivistic cultures where, e.g., there is strong emphasis on obedience to parental rules and less emphasis on the development of autonomy than in individualistic cultures such as the Dutch culture. For example, effects of parental behavioral control on internalizing and externalizing problems have shown to differ across cultures, probably due to cultural differences in the extent to which parents are expected to exert behavioral control in adolescence (Rothenberg et al., 2020). Moreover, the prevalence of problematic social media use in the Netherlands is relatively low (Boer et al., 2022b). In countries with high prevalence rates (e.g., some countries in Asia and the Middle East where the prevalence of problematic Internet use is generally higher than in European countries; Lozano-Blasco et al., 2022; Meng et al., 2022), problematic Internet use may have been normalized (Boer et al., 2020b). Because of this, it is possible that an increase in problematic social media use symptoms does not (strongly) impact parental restrictive mediation in such countries.

Fifth, motivations behind reactive restrictions are not captured in this study. There can be various reasons why parents intervene in their child’s ongoing Internet use. For instance, for some parents or in some situations applying reactive restrictions may be a way of regulating adolescents’ Internet use without establishing clear rules beforehand. For other parents or in other situations they may ask their child to turn off the device to enforce a predetermined rule, such as no screen use just before bedtime. Capturing the reasons behind reactive restrictions in future research may provide valuable insights.

## Conclusion

Restrictive mediation is one of the strategies parents apply with the idea to counteract possible negative consequences of media use, including problematic social media use. However, parental mediation strategies may also have unintended effects and may not only have an effect, but can also be affected by children’s (online) behavior. This study provided more insight in the possible effectiveness of two restrictive mediation practices in limiting adolescent problematic social media use by examining bidirectional within-family effects, while accounting for any potential confounding stable characteristics. The results suggest that problematic social media use affects parental restrictive mediation, but is not affected by these parenting practices. More specifically, findings suggest that parents appear to respond to signs of problematic social media use in their children by increasingly intervening in their ongoing Internet use, while, at the same time, they seem to give up on setting clearly defined rules. More research is needed on how parents can effectively limit problematic social media use among adolescents by, among others, testing possible moderators and testing other forms of parental mediation.
