# Correction to: Bidirectional Within-Family Effects of Restrictive Mediation Practices and Adolescents’ Problematic Social Media Use

**DOI:** 10.1007/s10964-024-02035-1

**Published:** 2024-06-17

**Authors:** Suzanne M. Geurts, Helen G. M. Vossen, Regina J. J. M. Van den Eijnden, Ina M. Koning

**Affiliations:** 1 Interdisciplinary Social Science, Utrecht University, Padualaan 14, 3584 CH Utrecht, The Netherlands; 2 Education and Pedagogy, Utrecht University, Heidelberglaan 1, 3584 CS Utrecht, The Netherlands; 3 Educational and Family Studies, VU Amsterdam, Van der Boechorststraat 7, 1081 BT Amsterdam, The Netherlands

Correction to: Journal of Youth and Adolescence (2024) 10.1007/s10964-024-01990-z

In the original publication of the article, the author noticed the errors in Ethical Approval, Funding and Authors’ Contributions. The updated texts have been presented with this erratum.

**Ethical Approval**

The study procedures have been approved by the Ethics Committee of the Faculty of Social and Behavioral Science at Utrecht University (FETC16-076).

**Funding**

The author(s) received no funding for this work.

**Authors’ Contributions**

S.M.G. participated in the design of the present study, performed statistical analysis, interpreted the data and drafted the manuscript; H.G.M.V. participated in the design of the present study and interpretation of the data and provided feedback on the manuscript; R.J.J.M.E. initiated and coordinated the data collection from the present study, participated in the design of the present study and interpretation of the data and provided feedback on the manuscript; I.M.K. participated in the design of the present study and interpretation of the data and provided feedback on the manuscript. All authors approved of the final version of the manuscript.

The original article has been corrected.

